# Multi-Objective Optimization in Ultrasonic Polishing of Silicon Carbide via Taguchi Method and Grey Relational Analysis

**DOI:** 10.3390/ma16165673

**Published:** 2023-08-18

**Authors:** Xin Chen, Shucong Xu, Fanwei Meng, Tianbiao Yu, Ji Zhao

**Affiliations:** 1 School of Mechanical Engineering and Automation, Northeastern University, Shenyang 110819, China; northeastern_cx@163.com (X.C.); mengfanwei6699@163.com (F.M.); 2 School of Materials Science and Engineering, Harbin Institute of Technology, Harbin 150001, China; xushucong1995@163.com

**Keywords:** ultrasonic polishing, silicon carbide, multi-objective optimization, grey relational analysis, material removal rate, surface roughness

## Abstract

As high-level equipment and advanced technologies continue toward sophistication, ultrasonic technology is extensively used in the polishing process of difficult-to-process materials to achieve efficiently smooth surfaces with nanometer roughness. The polishing of silicon carbide, an indispensable difficult-to-machine optical material, is extremely challenging due to its high hardness and good wear resistance. To overcome the current silicon carbide (SiC) ultrasonic polishing (UP) process deficiencies and strengthen the competitiveness of the UP industry, the multi-objective optimization based on the Taguchi–GRA method for the UP process with SiC ceramic to obtain the optimal process parameter combination is a vital and urgently demanded task. The orthogonal experiment, analysis of variance, grey relational analysis (GRA), and validation were performed to optimize the UP schemes. For a single objective of roughness and removal rate, the influence degree is abrasive size > preloading force > abrasive content > spindle speed > feed rate, and spindle speed > abrasive size > feed rate > preloading force > abrasive content, respectively. Moreover, the optimal process combination integrating these two objectives is an abrasive content of 14 wt%, abrasive size of 2.5 μm, preloading force of 80 N, spindle speed of 8000 rpm, and feed rate of 1 mm/s. The optimized workpiece surface morphology is better, and the roughness and removal rate are increased by 7.14% and 28.34%, respectively, compared to the best orthogonal group. The Taguchi–GRA method provides a more scientific approach for evaluating the comprehensive performance of polishing. The optimized process parameters have essential relevance for the ultrasonic polishing of SiC materials.

## 1. Introduction

Ultrasonic polishing is a high-performance surface finishing technology that utilizes nanoscale ultrasound-assisted subtractive manufacturing to achieve smooth surfaces with nanometer roughness and high-precision geometries [1,2]. The process involves the use of free abrasives to flatten the material surface under the integrated motion of rotation, ultrasonic vibration, and movement of the polishing pad, potentially avoiding a host of undesirable effects such as preventing the agglomeration of the polishing abrasives, increasing the material removal rate (MRR) from the polished area, and reducing the wear of the tools [1,3,4]. The resulting brilliant, mirror-like surfaces have become a popular alternative for a wide range of industrial applications such as semiconductor, biomedical, automotive, aerospace, and marine fields [5,6].

As high-level equipment and advanced technologies continue toward sophistication, the market for ultrasound-assisted components is being progressively expanded, and their affordability is being continuously driven down [7]. Currently, an economical ultrasound system component can be purchased for USD 20,000, which is a more economical choice compared to expensive equipment such as femtosecond lasers or magnetorheology [8]. Most of the pioneering scholars in this field have explored the combination of ultrasonic vibration technology with different polishing for a more systematic and comprehensive development of ultrasonic polishing technology. Shiou et al. [9] developed an ultrasonic-assisted spherical polishing system to investigate the effect of ultrasonic vibration on the polishing rate and surface quality of STAVAX stainless mold steel as well as the wear of the tool. The roughness was improved from 100 nm to 36 nm and the wear rate of polishing tools was reduced by 72% compared with the non-vibrated polishing process. Tsai et al. [10] utilized an ultrasonic-assisted chemical mechanical polishing system for the ultraprecision copper substrate finishing. They reported that the surface roughness with ultrasonic polishing was 1.448 nm, which was better than that recorded without ultrasonic polishing (2.378 nm). They also claimed that the ultrasonic vibration-induced chemical slurry dispersion contributed to the higher surface quality. Han et al. [11] employed a rectangle hexahedron ultrasonic sonotrode to conduct the polishing process on austenitic stainless steel. The results indicated that the horizontal vibration of the workpiece can improve the roughness of the polished surface, reduce polishing force, and reinforce the proportion of the plastic shear effect. Yu et al. [12] proposed an axial ultrasonic-assisted polishing method for the machinability of nickel-based alloy Inconel718. The corresponding analysis results showed that the introduction of an ultrasonic field changes the motion state and trajectory of the abrasive particles, thus effectively enhancing the polishing quality compared with traditional polishing, and the surface roughness can be reduced from the original 0.2 μm to about 6.5 nm in the ultrasonic vibration of 8 μm. All of the above scholars have studied ultrasonic polishing of metallic materials; in addition, for non-metallic hard and brittle materials, ultrasonic polishing research is still insufficient. Chu et al. [13] performed an ultrasonic polishing process for K9 optical glass. The results revealed that ultrasonic polishing can improve abrasive particle distribution uniform and polishing efficiency, in which the polishing efficiency was increased from 0.586 μm/min to 0.748 μm/min, and the surface roughness was promoted from 1.3175 nm to 0.9064 nm. Zhai et al. [14] conducted ultrasonic vibration-assisted magnetorheological polishing (UAMP) experiments to study the material removal of sapphire wafers. The material removal rate increased by approximately 3.4 times compared to conventional polishing. The largest MRR of 1.974 μm/h was achieved by UAMP. Zhang et al. [15] investigated the surface quality model of alumina ceramics processed by ultrasonic polishing at different ultrasonic amplitudes. The research results showed that the smaller ultrasonic amplitude (4 μm) leads to better roughness (41.09 nm), whereas the larger ultrasonic amplitude (8 μm) results in poorer roughness (56.27 nm) compared to ordinary polished surface roughness (48.18 nm) without ultrasonic polishing. Liu et al. [16] applied elliptical ultrasonic polishing on monocrystalline silicon. The research showed that the removal rate was increased by two times compared with ordinary polishing, and the roughness was reduced from 27.6 nm to 10.6 nm.

Over the past few years, we have been committed to the research of ultrasonic polishing. In our earlier research [17,18], a method of ultrasonic polishing coupled with chemical effects on a self-built 5-axis linkage polishing machine was proposed. Previous polishing studies for the difficult-to-machine characteristics of silicon carbide have shown that the ultrasonic and chemical factors are only a secondary effect, and that the mechanical effect still occupies the main function. Silicon carbide is an essential optical material in industrial production owing to its light mass, high space stability, and favorable specific stiffness [19,20,21,22,23]. It is widely used in the fields of semiconductors, precision bearings, and optical mirrors, especially in the manufacturing of optical system components with an exceptional precision surface finish [5,6]. However, the high hardness and good wear resistance of silicon carbide cause its polishing to be extremely challenging in the application process [24]. Therefore, this paper aims to explore the application of ultrasonic polishing technology in finishing silicon carbide optics to meet the requirements of an in-depth investigation of the silicon carbide polishing process and mechanism, and how to overcome current silicon carbide ultrasonic polishing field process deficiencies and strengthen the competitiveness of the ultrasonic polishing industry.

The Taguchi method has been widely applied in the design of experimental and data analysis of machining to explore the various factors and levels because it reduces the human trial and error process, avoids waste of resources and time, reduces the defect rate and loss rate of products, and improves the quality, reliability, and stability of products [25]. Yao et al. [26] experimentally investigated the effects of factors (e.g., loading force, abrasive concentration, and grain size) on the MRR and surface roughness of the cylindrical polishing of AISI 52100 steel with free abrasives using the Taguchi method. Wang et al. [27] used the Taguchi method to experimentally investigate the grinding process on AISI 1045 steel, and they studied the effects of grinding parameters and lubrication conditions on the combined technical, economic, health, and environmental benefits. Its advantage is to reduce processing time and costs by selecting a reasonable orthogonal array. Therefore, the optimization process in this study is based on the Taguchi method.

In this paper, the optimization objectives were presented firstly as polishing efficiency and surface quality. The analysis of variance (ANOVA) was performed to explore the effect of the selected parameters including abrasive content, abrasive size, preloading force, spindle speed, and feed rate on the optimization objectives. A grey relational analysis (GRA) method based on Taguchi analysis was used to obtain the appropriate parameters that can simultaneously satisfy the polishing response characteristics. The GRA analysis was further used to decide the weight of each objective response and convert all the responses into a single target. Finally, the orthogonal experiment analysis results were discussed, and the optimal scheme was further determined and verified. The optimized processing parameters have essential reference values for the ultrasonic polishing of SiC optical components.

## 2. Materials and Methods

### 2.1. Consumables and Equipment

The workpiece used in polishing is SiC ceramic (purchased from Hao Cai New Material Technology Co., Ltd., Foshan, China) with dimensions of 50 mm × 50 mm × 8 mm, and a chemical composition in weight percentage of Si 53.1%, C 43.8%, and other impurities elements in balance. Prior to polishing experiments and subsequent examinations, the SiC workpieces were cleaned in ultrasonic cleaning equipment for 10 min in anhydrous ethanol to remove any surface contaminants.

**Figure 1:**
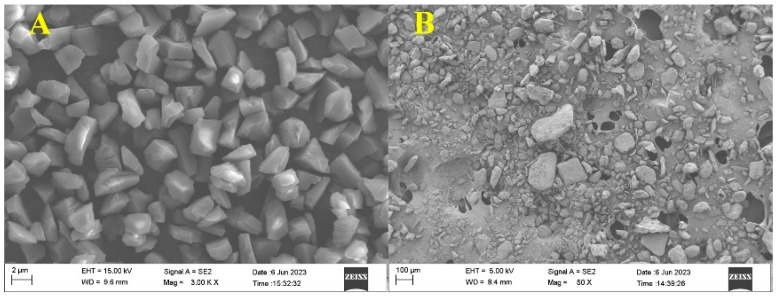
SEM microstructure of diamond abrasive (**A**) and suspension powder (**B**).

**Figure 2:**
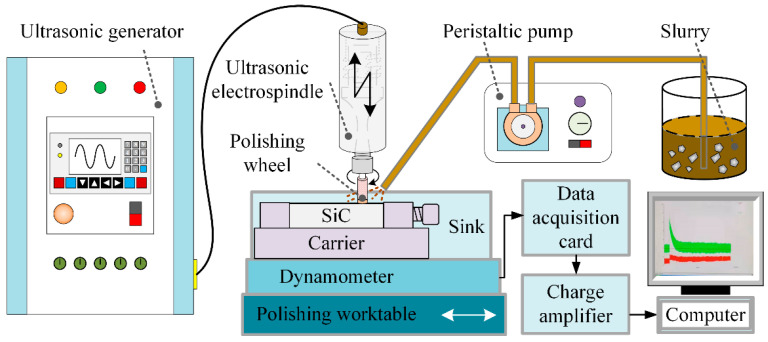
Schematic of ultrasonic polishing system.

For the ultrasonic polishing process, diamond abrasive suspension, consisting of diamond abrasive, suspension powder, and deionized water, was used due to its excellent removal, flow characteristics, and economic properties. Figure 1 shows the scanning electron microscopy (SEM) microstructure of diamond abrasive with the size of 3.5 μm and suspension powder. Prior to the preparation of the polishing suspensions, the two powders need to be dried in a drying oven at 80 °C and mixed properly in a blender. Figure 2 shows the schematic of ultrasonic polishing system. The system mainly consists of an ultrasonic electrospindle, a polishing suspension, and a reciprocating moving worktable. In addition, a polyurethane cylindrical polishing tool was fixed onto the bottom of the ultrasonic electrospindle thanks to the soft nature of elastomer, thereby facilitating the machining of smooth surfaces. An ultrasonic generator delivers an axial simple harmonic ultrasonic vibration with a frequency of 25 kHz and an amplitude of 10 μm to the polishing tool. A Kistler force measurement system, mainly including a computer, a three-component dynamometer, a computing A/D data conversion board, and a charge amplifier was employed for measuring and calibrating the polishing forces. The material properties of SiC ceramic, diamond abrasive, and polyurethane are displayed in Table 1.

**Figure 3:**
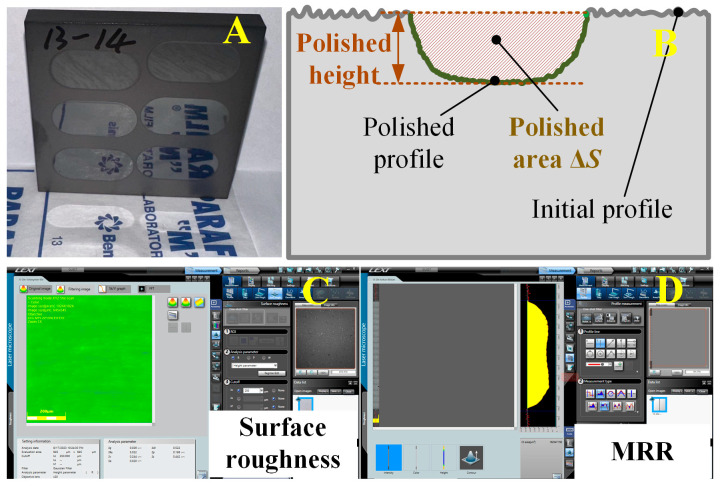
(**A**) Actual polished workpiece morphology, (**B**) cross-section diagram and measurement principle of surface roughness (**C**), and removal rate (**D**).

After ultrasonic polishing, the actual polished surface and the specific cross-section morphology were presented in Figure 3A,B. A laser scan confocal microscope (LSCM, OLS4100) was used to measure the surface topography, surface roughness *Sa*, and material removal rate, as shown in Figure 3C,D.

### 2.2. Taguchi Experimental Scheme and Implementation

Based on the previous studies [17,18], it was found that ultrasonic factors have less effect on polishing characteristics than the mechanical–physical factors. Therefore, the ultrasonic amplitude and frequency were fixed to 10 μm and 25 kHz, respectively. Abrasive content, abrasive size, preloading force, spindle speed, and feed rate were selected as the dominant factors to optimize the ultrasonic polishing process. Each dominant factor variable was controlled for five levels. Considering the general full factorial design of the experiments, 625 sets of experiments have to be conducted to evaluate the variation patterns of the polishing characteristics for these five aforementioned parameters at five levels. However, Taguchi method can optimize design parameters and meaningfully reduce the total number and duration of experiments [25], thereby reducing experiment costs to solve the problems of full factorial experiments without too much loss in overall information of the experimental data [28,29,30]. Therefore, the effects of the above five parameters on the polished SiC ceramic’s surface roundness and material removal rate were investigated by the Taguchi method. In this study, the total number of experiments designed using the Taguchi method was significantly reduced to only 25 using an L25(5^5^) Taguchi orthogonal experimental design with five factors and five levels, as listed in Table 2, in which parameters *A*, *B*, *C*, *D,* and *E* stand for abrasive content, abrasive size, preloading force, spindle speed, and feed rate, respectively. Moreover, the other polishing parameters in this paper was summarized in Table 3.

### 2.3. Optimization Procedures

Achieving a model for the optimal parameter implementation of an ultrasonic polishing system is one of the most practical and essential issues. In this section, the aim is to find the optimal values of each processing parameter to comprehensively improve the processing efficiency and quality of ultrasonic polishing of silicon carbide ceramic. In this paper, the two most important indicators of polishing characteristics are recognized as objective response characteristics based on Taguchi test, which are material removal rate and surface roughness. In accordance with the requirements of the polishing process, the roughness of the polished surface is as small as possible, while the material removal rate is as large as possible. Moreover, the grey relational analysis based on grey system theory is a method to measure the correlation between different factors based on the grey relational grade [31]. Its basic idea is to transform the discrete behavioral values of factors into segmented continuous lines and then construct a model to measure the correlation degree based on the geometric characteristics of the lines [32,33]. The closer the geometry of the lines stands for a greater correlation between the corresponding factors. Moreover, the GRA is widely used in discrete sequences for correlation analysis dealing with uncertainty, and multifactorial and discrete data, and this paper also aims to find the correlation degree between the systematic response of the polishing characteristics and the polishing parameters. Considering this situation, the GRA technique was introduced into the multi-objective analysis of Taguchi experiment to determine the weight of each objective response and convert all responses into a single target. Overall, the specific procedures are as follows:

Step 1: Calculating the SNRs.

The signal-to-noise ratio (SNR) is an evaluation metric for response characteristics that are subject to external interference. The SNR value can indicate the dispersion degree of the measured data around a nominal or target value, which can be modeled using a loss function. Generally, a higher SNR value means a better corresponding response characteristic. However, for the original polishing characteristic response itself, the two different responses exhibit opposite evaluation requirements; i.e., in polishing, the greater the material removal rate is better, while the smaller the surface roughness or smoother surface quality is better. Therefore, in order to make different evaluation criteria for different original response features, the loss function of the SNR method based on the properties of optimization proposes three categories [31,34,35,36]: namely, Larger-the-Better (LTB), Smaller-the-Better (STB), and Nominal-the-Better (NTB), as expressed by Equations (1)–(3). The LTB means that the larger original response characteristic is better, the STB means that the smaller original response characteristic is better, and the NTB means that the closer the original characteristic is to the target value the better. Thus, the LTB function is used for material removal rate, and the STB function is used for surface roughness.

(1) $$\mathrm{SNR}=-10\mathrm{lg}(\frac{1}{n}\sum_{i=1}^{n}\frac{1}{y_{i}{}^{2}})\;\mathrm{LTB}$$

(2) $$\mathrm{SNR}=-10\mathrm{lg}(\frac{1}{n}\sum_{i=1}^{n}y_{i}{}^{2})\;\mathrm{STB}$$

(3) $$\mathrm{SNR}=-10\mathrm{lg}(\frac{1}{n}\sum_{i=1}^{n}{\left(y_{i}-m\right)}^{2})\;\mathrm{NTB}$$

where *n* is the total number of experiments, *i* is 1~*n*, *y_i_* is one of the original target response characteristic eigenvalues in the *i*th experiment, and *m* is the expected value of the original target response characteristic eigenvalue of *y_i_*.

Step 2: Normalizing the SNRs.

The original data of SNRs need to be normalized to eliminate the scale effects of different response targets, which can be obtained as [37],

(4) $$x_{i}(j)=\frac{y_{i}(j)-\min y_{i}(j)}{\max y_{i}(j)-\min y_{i}(j)}$$

where *j* represents the target type, *y_i_*(*j*) is the raw SNR of the target response, and *x_i_*(*j*) is the normalized SNR of the target response. max *y_i_*(*j*) and min *y_i_*(*j*) represent the maximum and minimum values of the raw SNR.

Step 3: Calculating the grey relational coefficient (GRC).

The grey relational coefficient indicates the relationship between the ideal and actual experimental results for each column [38]. After the data pre-processing, the normalized SNR was used to calculate the grey correlation coefficient (GRC), calculated as [37],

(5) $$\zeta_{i}(j)=\frac{\underset{i}{\min}\underset{j}{\min}\left|x_{0}(j)-x_{i}(j)\right|+\eta \underset{i}{\max}\underset{j}{\max}\left|x_{0}(j)-x_{i}(j)\right|}{\left|x_{0}(j)-x_{i}(j)\right|+\eta \underset{i}{\max}\underset{j}{\max}\left|x_{0}(j)-x_{i}(j)\right|}$$

where *ζ_i_*(*j*) is the grey relational coefficient, *x*_0_(*j*) is the ideal value of *x_i_*(*j*), *x*_0_(*j*) = 1, and *η* is the distinguishing coefficient, ranging between 0 and 1. In this paper, *η* is set as 0.5 considering the moderate effect and stability [39]. $\underset{i}{\min}\underset{j}{\min}\left|x_{0}(j)-x_{i}(j)\right|$ and $\underset{i}{\max}\underset{j}{\max}\left|x_{0}(j)-x_{i}(j)\right|$ are the minimal and maximal values of the distance from *x*_0_(*j*) to *x_i_*(*j*).

Step 4: Calculating the response weight and grey relational grade (GRG).

The grey relational grade (GRG) is the weighted sum *ζ_j_* of the response feature GRC and its corresponding contribution *ω_j_*, can be obtained as [27],

(6) $$\mathrm{GRG}=\sum_{j=1}^{k}\omega_{j}\zeta_{j}$$

(7) $$\zeta_{j}=\frac{1}{n}\sum_{i=1}^{n}\zeta_{i}(j)\;\;\;j\;=\;1,\;2,\ldots ,\;k$$

(8) $$\omega_{j}=\frac{\zeta_{j}}{\sum_{j=1}^{k}\zeta_{j}}\;\;\;j\;=\;1,\;2,\ldots ,\;k$$

where *k* is the total number of target responses.

**Figure 4:**
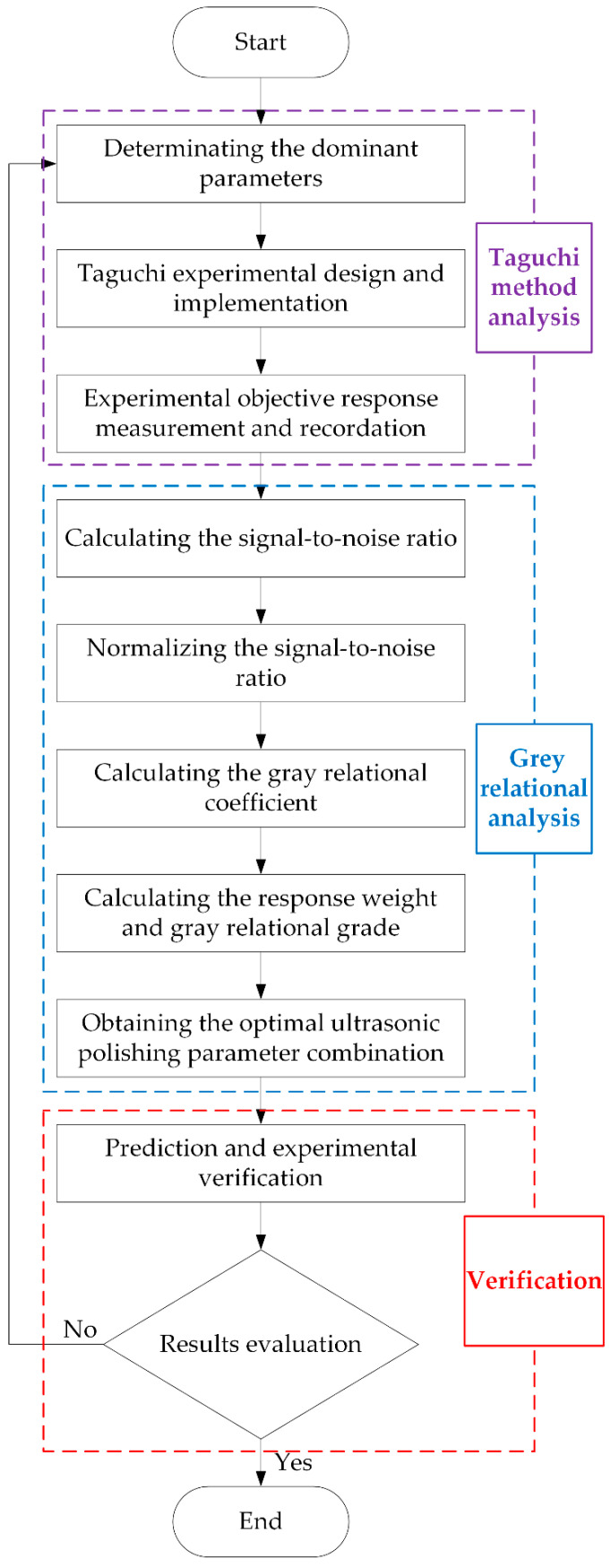
Flowchart of Taguchi–GRA optimization method.

As shown in Figure 4, the flowchart of the Taguchi–GRA optimization method was given.

## 3. Results and Discussion

### 3.1. Effect of Process Parameters on Response Characteristics

**Figure 5:**
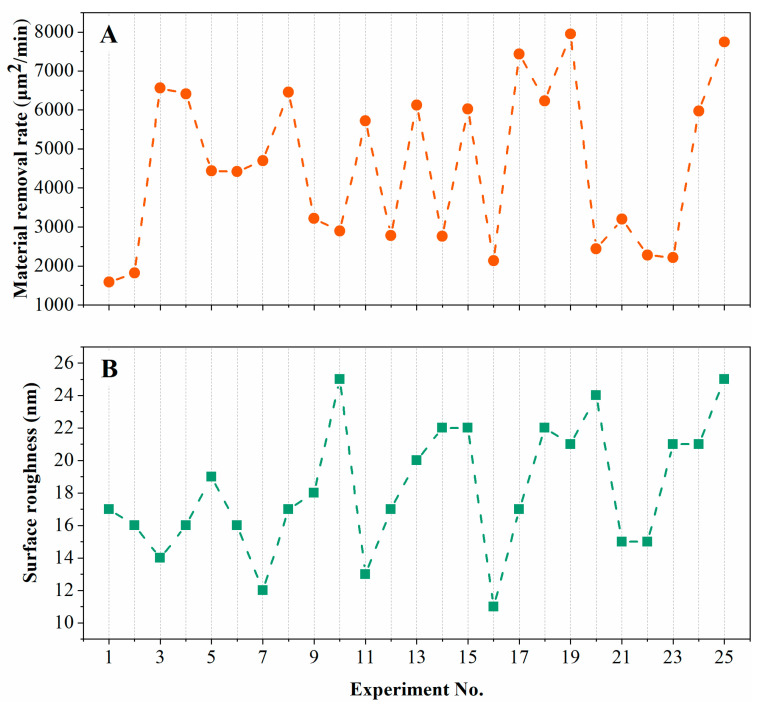
(**A**) Material removal rate and (**B**) surface roughness results of Taguchi orthogonal experiment.

The calculated results of the material removal rate and surface roughness response characteristics of the orthogonal experiments after laser confocal microscopy inspection are summarized in Table 4 and Figure 5.

**Figure 6:**
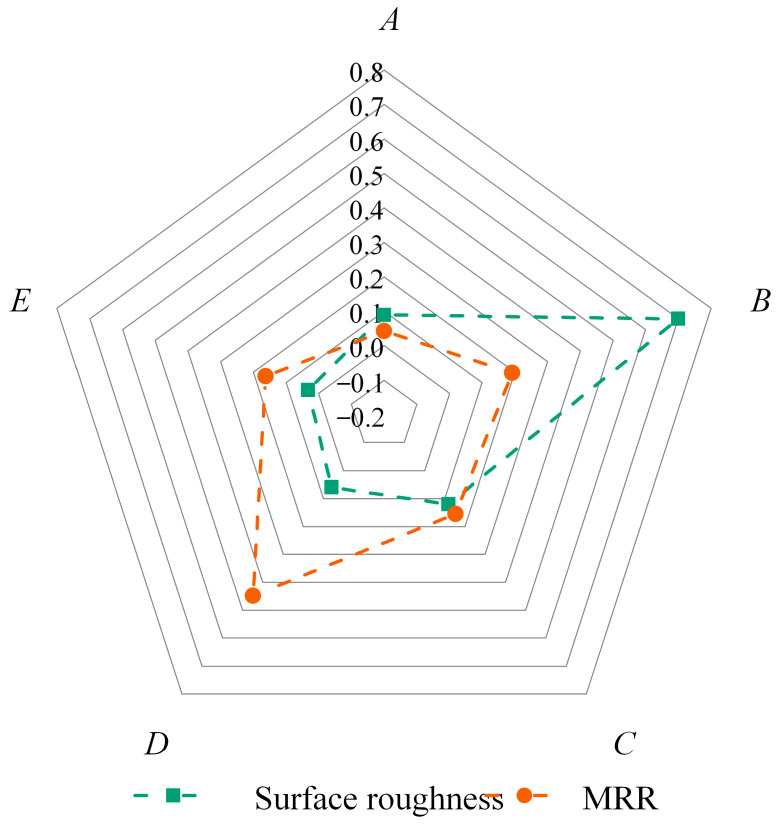
Radial diagram of contributions of selected parameters in surface roughness and MRR. Note that the selected parameters *A*, *B*, *C*, *D* and *E* stand for abrasive content, abrasive size, preloading force, spindle speed and feed rate, respectively.

**Figure 7:**
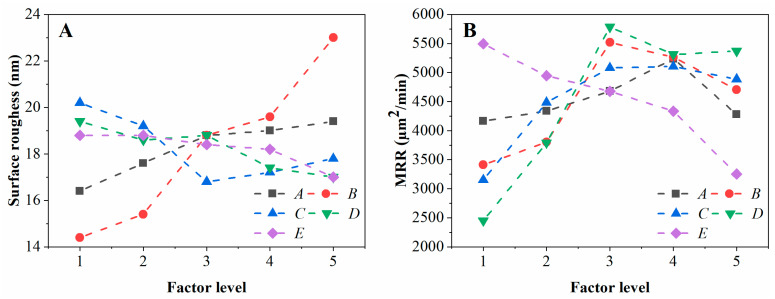
Main effects plot of the mean value of surface roughness (**A**) and material removal rate (**B**) at different parameters. Note that the selected parameters *A*, *B*, *C*, *D* and *E* stand for abrasive content, abrasive size, preloading force, spindle speed and feed rate, respectively.

In order to explore the relative contribution and effect of the selected parameters on the results, the analysis of variance (ANOVA) was performed to calculate the adjusted sum of squares of deviation (Adj SS), adjusted mean squares (Adj MS), and F-value using MINITAB 19 software. Table 5 shows the ANOVA results of surface roughness, indicating that only the F-value of the abrasive size is 7.31, showing a significant effect on the surface roughness. Compared to the F-value of abrasive size, the F-values of the other four factors ranged from 0.34 to 1.25, which illustrates the fact that the effect of abrasive size on surface roughness is much greater than that of the other four factors. In addition, the contribution of five factors to roughness was calculated based on the F-value, as displayed in Table 5 and Figure 6. Meanwhile, the contribution rate of the abrasive content, abrasive size, preloading force, spindle speed, and feed rate to the surface roughness is 8.90%, 69.95%, 11.96%, 5.93%, and 3.25%, respectively. Hence, for the surface roughness, the most significant UP parameter is the abrasive size, followed in order by the preloading force, abrasive content, and spindle speed, whereas the least influential parameter is the feed rate. Furthermore, Figure 7 shows the detailed distribution of the surface roughness at different polishing parameters. With the increasing abrasive content and abrasive size, the surface roughness increases. The deeper scratches left on the SiC surface by more and larger abrasives will increase the surface roughness [40]. As the preloading force increases, the surface roughness first decreases and then increases, probably because the large preloading force leads to the deep indentation of abrasive into the SiC workpiece surface [26]. Meanwhile, the surface roughness basically decreases with the increase in spindle speed and feed rate. On the one hand, the higher polishing speed increases the centrifugal effect of the polishing solution, which increases the chance of contact between the soft polishing pad and the hard SiC surface, and reduces the number of scratches produced by the abrasive on the SiC surface. On the other hand, the increased speed increases the friction of the abrasive per unit of time in the contact area of the surface of the workpiece, and the heat generated is transferred to the polishing solution, resulting in a slight softening of the workpiece surface at higher temperatures and, thus, reducing the roughness. Therefore, for surface roughness, the optimal combination of polishing parameters in this paper is *A*_1_*B*_1_*C*_3_*D*_5_*E*_5_, i.e., abrasive content of 2 wt% (level 1), abrasive size of 0.5 μm (level 1), preloading force of 60 N (level 3), spindle speed of 14,000 rpm (level 5), and feed rate of 3 mm/s (level 5), as shown in Figure 6.

Table 6 shows the ANOVA results of the material removal rate, indicating that only the F-value of the spindle speed is 2.52, showing a relatively significant effect on the material removal rate. Compared to the F-value of spindle speed, the F-values of the other four factors ranged from 0.24 to 1.08, which illustrates the fact that the effect of spindle speed on material removal rate is much larger than that of the other four factors. Table 6 and Figure 6 further demonstrate the contribution of five factors to roughness. It can be found that the contribution rate of the abrasive content, abrasive size, preloading force, spindle speed, and feed rate to the material removal rate is 4.27%, 19.22%, 15.48%, 44.84%, and 16.19%, respectively. Hence, for the material removal rate, the most significant UP parameter is the spindle speed, followed in order by the abrasive size, feed rate, and preloading force, whereas the least influential parameter is the abrasive content. Meanwhile, Figure 7B shows the detailed distribution of the material removal rate at different polishing parameters. Neglecting the external interference or some errors in the experiment itself, it can be seen from Figure 7B that with the increase in abrasive content, abrasive size, and preloading force, the material removal rate basically increases first and then decreases. Within a reasonable range, the increase in these three factors increases the removal depth of individual abrasives on the workpiece and the overall number of abrasives between the polishing pad and the workpiece to a certain extent, which, in turn, increases the removal rate. However, an inflection point in the MRR occurred when the abrasive content, abrasive size, and preloading force were at the 4th, 3rd, and 4th levels, respectively. This may be because the larger-sized diamond abrasive will lead to a reduction in the number of active abrasives in the contact area, the higher concentration of diamond abrasive content will definitely lead to poor fluidity of the configured suspension, and the higher pressure will lead to a larger deformation and compression of the polishing pad, which will reduce the intrusion of the polishing fluid into the contact area, reduce the scratches of the consolidated abrasive, and also reduce the space for the presence of free abrasive, which will lead to a weakened ultrasonic cavitation effect, ultimately resulting in a decrease in material removal. In addition, when the spindle rotation speed increases from 2000 rpm to 8000 rpm, the material removal rate shows a significant increase from 2449.16 μm^2^/min to 5783.21 μm^2^/min. However, when the rotation speed exceeds 8000 rpm, the material removal rate does not continue to increase and basically stabilizes in the range of 5311.46 μm^2^/min to 5368.22 μm^2^/min, probably because the centrifugal effect of the polishing liquid increases when the spindle rotation speed is higher, which, in turn, reduces the removal of diamond grain particles on silicon carbide. The increase in feed rate leads to faster wear of the polishing pads and reduced residence time of the diamond abrasive in the contact area, which may result in a decreasing material removal rate. Therefore, the optimal combination of polishing parameters for the material removal rate response in this paper is *A*_4_*B*_3_*C*_4_*D*_3_*E*_1_, i.e., abrasive content of 14 wt% (level 4), abrasive size of 2.5 μm (level 3), preloading force of 80 N (level 4), spindle speed of 8000 rpm (level 3), and feed rate of 1 mm/s (level 1), as shown in Figure 6.

### 3.2. Multi-Response Optimization

#### 3.2.1. Calculation of SNRs

Since the smaller surface roughness and larger material removal rate are better, we need to synthesize the two response characteristics to optimize the best process parameters. Table 7 shows the calculation results of SNR of response characteristics using Equations (1)–(3). The response characteristics SNR in Table 7 were normalized as raw data to give the two responses a common scale using Equation (4). Further, the normalized SNR data were substituted into Equation (5) to calculate the GRC, and then the acquired GRC data were substituted into Equations (6)–(8) to obtain the final GRG. It can be calculated from Equations (7) and (8) that the contribution of the normalized SNRs of surface roughness and material removal rate was 44.82% and 55.18%, respectively. The calculation results of the normalized SNRs, GRC, and GRG are summarized in Table 8.

#### 3.2.2. Calculation of GRC and Response Weights

**Figure 8:**
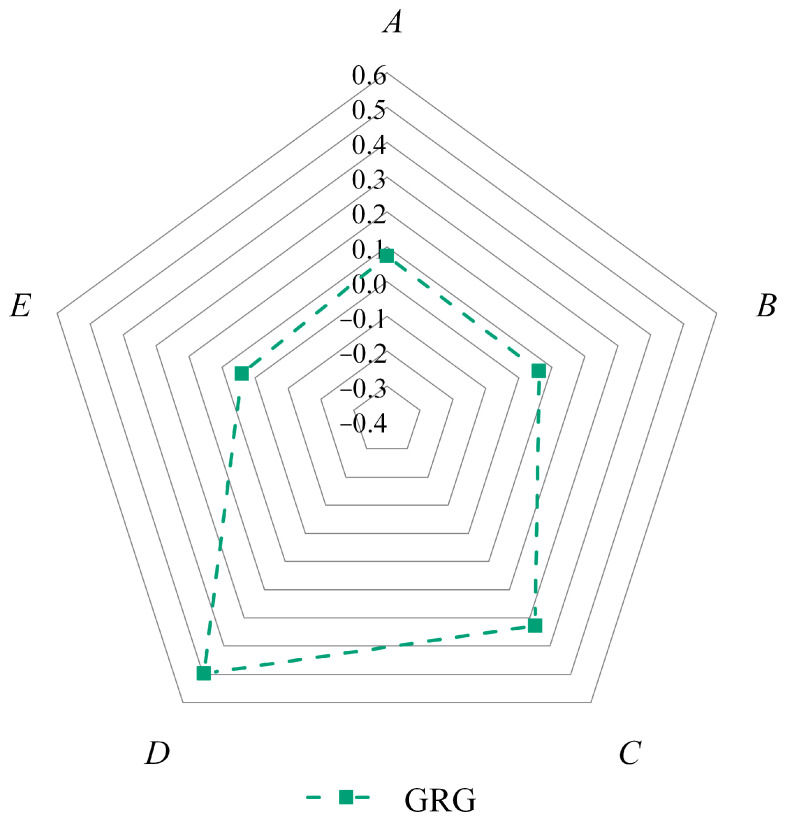
Radial diagram of contributions of selected parameters in GRG. Note that the selected parameters *A*, *B*, *C*, *D* and *E* stand for abrasive content, abrasive size, preloading force, spindle speed and feed rate, respectively.

**Figure 9:**
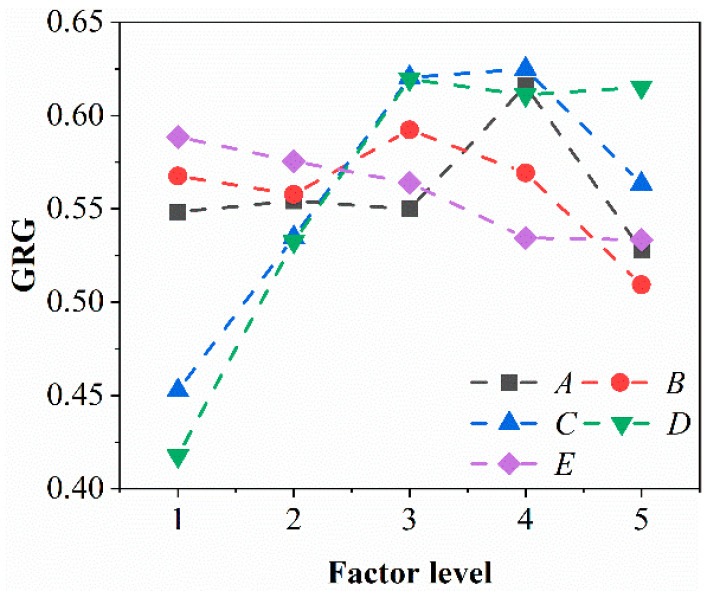
Main effects plot of the mean value of GRG at different parameters. Note that the selected parameters *A*, *B*, *C*, *D* and *E* stand for abrasive content, abrasive size, preloading force, spindle speed and feed rate, respectively.

The ANOVA results of GRG are listed in Table 9, indicating that the F-values of the preloading force and spindle speed are 1.66 and 2.51, respectively, showing a significant effect on GRG. Compared to the F-values of preloading force and spindle speed, the F-values of the other three factors ranged from 0.2 to 0.37, which illustrates the fact that the effect of preloading force and spindle speed on GRG is much greater than that of the other three factors. Meanwhile, the contribution rate of the abrasive content, abrasive size, preloading force, spindle speed, and feed rate to the surface roughness is 7.33%, 6.14%, 32.87%, 49.70%, and 3.96%, respectively, as shown in Table 9 and Figure 8. Hence, for the GRG, the most significant UP parameter is the spindle speed, followed in order by the preloading force, abrasive size, and abrasive content, whereas the least influential parameter is the feed rate. From Figure 9, GRG basically increases and then decreases with increasing abrasive content, abrasive size, preloading force, and spindle speed. As the feed rate increases, the material removal rate decreases. The results were obtained after the comprehensive evaluation, and the optimal combination of polishing parameters in this paper are *A*_4_*B*_3_*C*_4_*D*_3_*E*_1_, i.e., abrasive content of 14 wt% (level 4), abrasive size of 2.5 μm (level 3), preloading force of 80 N (level 4), spindle speed of 8000 rpm (level 3), and feed rate of 1 mm/s (level 1).

### 3.3. Optimal Combination Verification

The optimal process parameter combinations are not in the orthogonal experimental design table; therefore, we borrow GRG’s prediction equation 9 to determine the effectiveness of the multi-objective optimization results [41].

(9) $$GRG_{pre}=GRG_{mean}+\sum_{j=1}^{k}\left(GRG_{j}-GRG_{mean}\right)$$

where *GRG_mean_* is the mean value of all the GRG data. *GRG_j_* is the mean value of all the *GRG* data for the *j*th processing parameters at the selected optimal level, and *k* is the number of input factors (process parameters). In this case, *j* = 1, 2.

**Figure 10:**
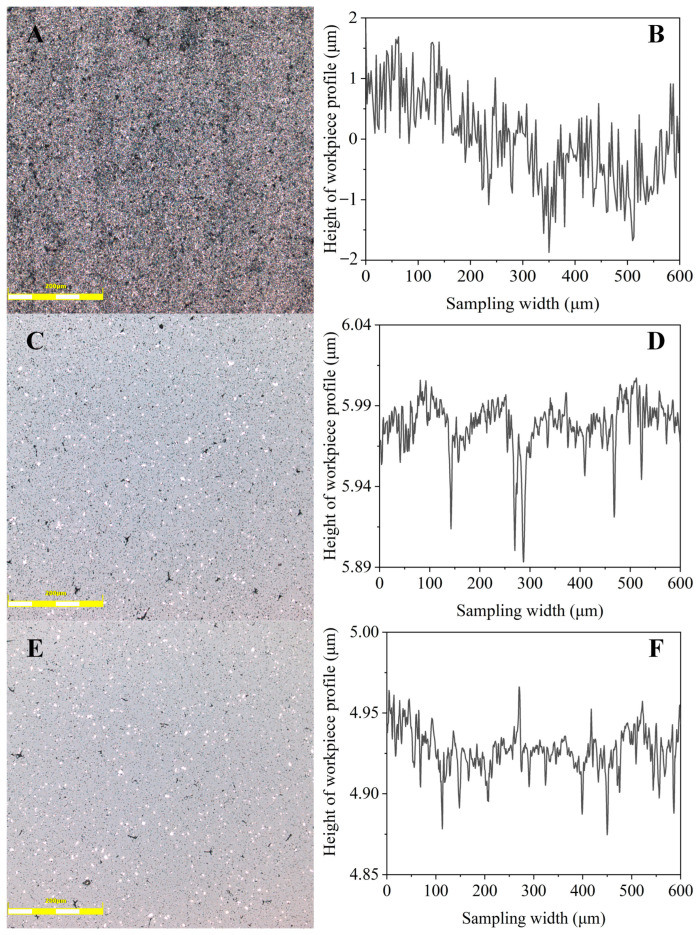
Microscopic surface morphology and surface roughness profile of as-received workpiece (**A**,**B**), No.3 workpiece at the L25 orthogonal experimental design (**C**,**D**), and optimal workpiece of validation experiment (**E**,**F**).

Substituting the GRG for the optimum combination of process parameters into Equation (9), the predicted value of GRG for the optimum combination is obtained as 0.8043. This is an improvement of 10.45% as compared to the maximum value of GRG in orthogonal experiments of 0.7282. This reveals the success of the optimized combination of process parameters as well as the improvement in the polishing response characteristics. Moreover, through further ultrasonic polishing experiments with optimized process parameters, the results show that the measured surface roughness and material removal rate are 13 nm and 8424.41 μm^2^/min, respectively, which were improved by 7.14% and 28.34%, respectively, compared to the experimental group with the best GRG in Table 8. And the optimized workpiece showed a better surface morphology, as shown in Figure 10. The LSCM image of the as-received workpiece surface in Figure 10A displays abundant tiny protrusions as well as some intergranular pores, and it is also verified in Figure 10B that the original surface is more undulating and still has a more fluctuating profile trajectory over a large height range of 4 μm, compared to the polished surface in Figure 10D,F. Moreover, after ultrasonic polishing, the LSCM images of Figure 10C,E show that the polished surface is bright white compared to the as-received surface, but there are still defects on the surface due to the intergranular pores. From Figure 10D,F, it can also be seen that the optimized surface exhibits more uniform and smaller undulations when the height range is controlled at the same 0.15 µm, which verifies the improvement in surface roughness and surface finish. Furthermore, in our previous study [17], the surface roughness was 17.6 nm in UP with an amplitude of 9 μm, and the optimization in the validation experiment resulted in an improvement of 26.13%, which also reveals the effectiveness of optimization.

## 4. Conclusions

Ultrasonic polishing is a highly effective method for polishing SiC optical components. In this paper, a Taguchi method was utilized to ascertain the relative contribution and effect of abrasive content, abrasive size, preloading force, spindle speed, and feed rate on surface roundness and material removal rate. Moreover, a multi-response optimization method, the GRA method based on a Taguchi analysis, was proposed to determine the appropriate and comprehensive ultrasonic polishing parameters. The main findings are as follows:

1. The analysis of variance was performed to investigate the effect of selected parameters on polishing characteristics. The influence degree on the surface roughness is abrasive size > preloading force > abrasive content > spindle speed > feed rate. The best process combination on the surface roughness is the abrasive content of 2 wt%, abrasive size of 0.5 μm, preloading force of 60 N, spindle speed of 14,000 rpm, and feed rate of 3 mm/s. The influence order on the material removal rate is spindle speed > abrasive size > feed rate > preloading force > abrasive content. The best process combination on the material removal rate is the abrasive content of 14 wt%, abrasive size of 2.5 μm, preloading force of 80 N, spindle speed of 8000 rpm, and feed rate of 1 mm/s.
2. The Taguchi–GRA optimization method was operated successfully, and the best process combination combining material removal rate and surface roughness is the abrasive content of 14 wt%, abrasive size of 2.5 μm, preloading force of 80 N, spindle speed of 8000 rpm, and feed rate of 1 mm/s. The optimized workpiece showed improvements in surface roughness and material removal rate by 7.14% and 28.34%, respectively, compared to the group with the best GRG.
3. The Taguchi–GRA method provides a more scientific approach for evaluating the comprehensive performance of precision polishing. The research findings have essential relevance for ultra-precision polishing of optical ceramic materials, especially silicon carbide.

## Figures and Tables

**Table 1:** The material properties of SiC ceramic, diamond abrasive, and polyurethane [15,17,26].

Properties	Material Type		
	SiC	Diamond	Polyurethane
Density (g/cm^3^)	3.12	3.52	0.49
Young’s modulus (GPa)	410	1000	2.29 × 10^−3^
Poisson ratio	0.16	0.07	0.47
Hardness (GPa)	28.4–33.2	50	72 (Shore A)
Fracture toughness (Mpa·m^1/2^)	4.5	-	-

**Table 2:** Taguchi Orthogonal experiment run design.

No.	Factors				
	*A*: Abrasive Content *W*_a_ (wt%)	*B*: Abrasive Size *D*_a_ (μm)	*C*: Preloading Force *F*_0_ (N)	*D*: Spindle Speed *n* (rpm)	*E*: Feed Rate *v*_w_ (mm/s)
1	2	0.5	20	2000	1
2	2	1.5	40	5000	1.5
3	2	2.5	60	8000	2
4	2	3.5	80	11,000	2.5
5	2	7	100	14,000	3
6	6	0.5	40	8000	2.5
7	6	1.5	60	11,000	3
8	6	2.5	80	14,000	1
9	6	3.5	100	2000	1.5
10	6	7	20	5000	2
11	10	0.5	60	14,000	1.5
12	10	1.5	80	2000	2
13	10	2.5	100	5000	2.5
14	10	3.5	20	8000	3
15	10	7	40	11,000	1
16	14	0.5	80	5000	3
17	14	1.5	100	8000	1
18	14	2.5	20	11,000	1.5
19	14	3.5	40	14,000	2
20	14	7	60	2000	2.5
21	18	0.5	100	11,000	2
22	18	1.5	20	14,000	2.5
23	18	2.5	40	2000	3
24	18	3.5	60	5000	1
25	18	7	80	8000	1.5

**Table 3:** The other polishing parameters in this paper [18].

Parameters	Value
Ultrasonic amplitude *A* (μm)	10
Ultrasonic frequency *f* (kHz)	25
Polishing tool diameter *D*_t_ (mm)	10
Polishing time *t* (min)	20
Polishing distance *d* (mm)	10

**Table 4:** Response characteristics results of Taguchi orthogonal experiment.

No.	Response Characteristics	
	Surface Roughness (nm)	Material Removal Rate (μm^2^/min)
1	17	1588.15
2	16	1824.73
3	14	6564.07
4	16	6408.33
5	19	4442.52
6	16	4418.63
7	12	4702.45
8	17	6451.84
9	18	3219.67
10	25	2896.72
11	13	5716.37
12	17	2781.35
13	20	6126.36
14	22	2759.52
15	22	6020.24
16	11	2139.70
17	17	7432.72
18	22	6228.33
19	21	7947.56
20	24	2438.75
21	15	3197.98
22	15	2282.79
23	21	2217.88
24	21	5975.30
25	25	7741.11

**Table 5:** ANOVA results of surface roughness.

Source	DF	Adj SS	Adj MS	F-Values	Contribution
*A*	4	30.16	7.54	0.93	8.90%
*B*	4	238.16	59.54	7.31	69.95%
*C*	4	40.56	10.14	1.25	11.96%
*D*	4	20.16	5.04	0.62	5.93%
*E*	4	10.96	2.74	0.34	3.25%
Error	4	32.56	8.14	-	-
Total	24	372.56	-	-	-

**Table 6:** ANOVA results of material removal rate.

Source	DF	Adj SS	Adj MS	F-Values	Contribution
*A*	4	3,766,513	941,628	0.24	4.27%
*B*	4	16,589,875	4,147,469	1.08	19.22%
*C*	4	13,298,819	3,324,705	0.87	15.48%
*D*	4	38,784,722	9,696,180	2.52	44.84%
*©E*	4	13,965,735	3,491,434	0.91	16.19%
Error	4	15,373,706	3,843,427	-	-
Total	24	101,779,369	-	-	-

**Table 7:** Calculation results of SNR of response characteristics.

No.	SNR of Response Characteristics	
	Surface Roughness	Material Removal Rate
1	−24.6090	64.0178
2	−24.0824	65.2240
3	−22.9226	76.3435
4	−24.0824	76.1349
5	−25.5751	72.9526
6	−24.0824	72.9058
7	−21.5836	73.4465
8	−24.6090	76.1937
9	−25.1055	70.1562
10	−27.9588	69.2381
11	−22.2789	75.1424
12	−24.6090	68.8851
13	−26.0206	75.7441
14	−26.8485	68.8167
15	−26.8485	75.5923
16	−20.8279	66.6070
17	−24.6090	77.4230
18	−26.8485	75.8874
19	−26.4444	78.0047
20	−27.6042	67.7434
21	−23.5218	70.0975
22	−23.5218	67.1693
23	−26.4444	66.9188
24	−26.4444	75.5272
25	−27.9588	77.7761

**Table 8:** Calculation results of normalized SNR, GRC, and GRG.

No.	Normalized SNR		GRC		GRG
	Surface Roughness	Material Removal Rate	Surface Roughness	Material Removal Rate	
1	0.4698	0.0000	0.4853	0.3333	0.4015
2	0.5436	0.0862	0.5228	0.3537	0.4295
3	0.7063	0.8812	0.6299	0.8081	0.7282
4	0.5436	0.8663	0.5228	0.7890	0.6697
5	0.3343	0.6388	0.4289	0.5806	0.5126
6	0.5436	0.6354	0.5228	0.5783	0.5534
7	0.8940	0.6741	0.8251	0.6054	0.7039
8	0.4698	0.8705	0.4853	0.7943	0.6558
9	0.4001	0.4389	0.4546	0.4712	0.4638
10	0.0000	0.3732	0.3333	0.4437	0.3943
11	0.7965	0.7954	0.7108	0.7096	0.7101
12	0.4698	0.3480	0.4853	0.4340	0.4570
13	0.2718	0.8384	0.4071	0.7557	0.5995
14	0.1557	0.3431	0.3719	0.4322	0.4052
15	0.1557	0.8275	0.3719	0.7435	0.5770
16	1.00	0.19	1.0000	0.3803	0.6580
17	0.47	0.96	0.4853	0.9232	0.7270
18	0.16	0.85	0.3719	0.7676	0.5903
19	0.21	1.00	0.3883	1.0000	0.7259
20	0.05	0.27	0.3448	0.4053	0.3782
21	0.62	0.43	0.5696	0.4693	0.5143
22	0.62	0.23	0.5696	0.3923	0.4717
23	0.21	0.21	0.3883	0.3868	0.3875
24	0.21	0.82	0.3883	0.7384	0.5815
25	0.00	0.98	0.3333	0.9683	0.6837

**Table 9:** ANOVA results of GRG.

Source	DF	Adj SS	Adj MS	F-Values	Contribution
*A*	4	0.02216	0.00554	0.37	7.33%
*B*	4	0.01884	0.004709	0.31	6.14%
*C*	4	0.10021	0.025052	1.66	32.87%
*D*	4	0.15115	0.037787	2.51	49.70%
*E*	4	0.01211	0.003027	0.2	3.96%
Error	4	0.06027	0.015067	-	-
Total	24	0.36473	-	-	-

## Data Availability

Not applicable.
